# Some Facts—Chemical and Otherwise—About Dentifrices*Read at the annual meeting of the Massachusetts Dental Society, Boston, Mass., May 8-10, 1913.

**Published:** 1913-07-15

**Authors:** Howard C. Kelly

**Affiliations:** The Central High School, Springfield, Mass.


### SOME FACTS—CHEMICAL AND OTHERWISE—ABOUT DENTIFRICES.*

BY PROF. HOWARD C. KELLY, OF THE CENTRAL HIGH SCHOOL, SPRINGFIELD, MASS.

If the dentists of this commonwealth are so besieged with work, so overwhelmed, submerged and literally buried beneath a mass of decayed teeth that they cannot spare the time to put a little thought and energy into the prevention of diseased conditions, then I must heartily congratulate their pocketbooks, and suggest that a strong stimulant be applied to their common sense.

Are the dentists of Massachusetts more interested in disease than in health? Do they prefer to treat a diseased tooth rather than produce conditions which will render decay and suffering less common? Does their interest center in the cash they receive rather than in the aid they give? Are these things true? I dislike to believe it, but if so, it is certainly high time for a radical readjustment of our standards of value, and a new definition of the word "ethical." Such a readjustment must come if the dental profession is to maintain the standing it desires and deserves among the thinking people of every community.

If, on the other hand, our inferences are not true, how

*Read at the annual meeting of the Massachusetts Dental Society, Boston, Mass., May 8-10, 1913.

can we account for the general ignorance which exists, even among dentists, concerning a subject which those same dentists tell us is of the highest importance? I leave the answer to you.

But does such ignorance really exist? For some years I have been questioning, without previous warning, the pupils in the entering class of the Central High School of Springfield, Massachusetts, regarding the tooth preparations which they use, and the answers received are, it seems to me, rather uncomfortably good evidence that we have not overstated the case. These pupils are fairly representative of all parts of the city, and every class has yielded almost exactly like results. From this I conclude that the results are fairly representative of the average Massachusetts city. Take, for example, the results of this year's test.

The questions asked were four in number, and the following answers resulted:

1. *Do you use any sort of tooth preparation?*

| | |
|---|---|
| Total number reporting | 230 |
| Not using any preparation | 13 or 6% |
| Using some preparation | 217 or 94% |

These answers are practically identical year after year. The number responding varies, but the percentage of each remains about the same. The use of some sort of tooth preparation seems to be practically universal.

2. *Is the preparation you use powder, paste or liquid?*

| | |
|---|---|
| Paste | 102 or 47% |
| Powder | 96 or 44% |
| Liquid | 19 or 9% |
| | 217 |

The powders and pastes seem to be about equally popular at the present time, but the results of several years indicate that the pastes, probably on account of their greater convenience, are making rapid gains. This year, for the first time, they have passed the powders. The liquids, on the other hand, are just as steadily loosing ground.

*3. What is the name of the preparation you use?*

| | |
|---|---|
| Colgate's | 73 |
| Dr. Lyon's | 15 |
| Kolynos | 13 |
| Sanitol | 12 |
| Larkin's | 8 |
| Hydrogen peroxide | 5 |
| Miscellaneous | 12 |
| "I don't know" | 79 |
| | 217 |

Note particularly the last figures. Nearly 40 percent. of those answering had absolutely no idea what preparation they were using! The others require little comment. It is quite evident that Colgate's advertising has not been entirely in vain.

*4. Why do you use that particular kind?*

| | |
|---|---|
| Some reason given by | 85 |
| "I don't know" | 132 |
| | 217 |

These figures are illuminating. No less than 60 percent. of those using tooth preparations had no reason to venture for using the particular preparation which they did. They had not even the glimmer of an idea whether or why it might be better or worse than some others. To them a tooth preparation was a tooth preparation and would clean the teeth. To use something was the main object.

No less illuminating are the reasons given by those who did venture a reply to the question:

| | |
|---|---|
| Like the taste of it | 55 |
| Advised by a dentist | 19 |
| Have always used it | 5 |
| Widely advertised | 3 |
| Know the maker | 2 |
| High Price | 1 |
| | 85 |

To these I might add two which were received the year before. They were: "Prevents infantile paralysis" and "Ma makes me." It is interesting to note that of the 19 cases where some preparation was advised by a dentist, all but two were preparations which bore the dentists own name. Of the two exceptions to this rule, one was a preparation containing two ingredients which should never be found in any dentifrice, and the other was a preparation which the dentist said "must be all right or they wouldn't advertise it so much." Not one of the reasons, even that of the dentist himself, makes the slightest allusion to any property of the preparation which would indicate suitability to purpose, high quality or anything else which should govern a thinking person when choosing a dentifrice.

I believe that the foregoing is sufficient to show that there is at present in our State a condition of things which should make us the least bit ashamed of ourselves. Is it not evident that these young people, arrived at an age when they should be taking intelligent care of their teeth, had very little knowledge of the dentifrices they were using, and that the correctness of even that little knowledge was open to very serious question? Is it not clear that, while tooth preparations are used by nearly everyone, the general public has little or no knowledge of their composition, effects or real value?

If further proof is needed, it may be readily found in the number of different preparations on the market to-day. This number is almost beyond computation. There are literally hundreds, perhaps thousands, and new ones are springing up all the time. In one small drug store I counted 88 varieties, and I have no doubt there were a few others under the counter. These all sell for a good price, yet the cost of the basic material is very small. In more than one instance the container has been shown to cost more than the contents. The large demand, of which we have spoken, together with the immense profit, has produced a sharp competition among manufacturers to supply the drug store trade, and this has resulted in many worthless and some harmful preparations being thrown upon the market. The pity of it is that the worthless and even the harmful seem to enjoy as wide or wider popularity than do the desirable preparations.

The method of advertising these numerous preparations reflects the ignorance of the day. Seldom, if ever, is quality and suitability to purpose made prominent. A delightful flavor, a new container or something similar is the advantage urged. Also, those secondary advertising dodges, such as appearance of package, etc., are made much of. One powder is put up in an elaborate pressed glass bottle. It would be interesting to compare the cost of that bottle with the cost of the powder it holds. Does the elaborate bottle make the powder any better? Does it enable the manufacturer to serve the customer to better advantage? Hardly! The hope is that, without regard to what the powder may be, the purchaser will be impressed with the desirable appearance of the bottle, and will buy the *bottle*, taking the powder because it is impossible to get the bottle in any other way. That same bottle is about a quarter of an inch thick, which insures that the purchaser shall not get too much for his money. Another bottle has an exaggerated wasp-waist effect, another is diamond shaped, and another has a highly colored picture of an alleged beautiful young lady—all that the contents may be forgotten in the attractiveness of the container.

The dental profession must shoulder its full share of responsibility for the ignorance, misinformation and misrepresentation which these things imply. It is all very well to say, as one dentist did to me, that it is not your business, that the schools can and should incorporate it as a part of their work, but this shifting of responsibility does not meet the question. I agree that the schools should have their part in this work of education, but let there be some *dental* as well as public schools. Let our dentists be our instructors, and then we shall have a corps of teachers who can speak with authority and to whom the public will listen, as they will not to the layman.

The information most needed by the public at large is neither elaborate nor technical. Rather it is the simple, practical facts which appeal to their common sense, and give them a method of determining for themselves whether or not the use of any particular preparation is wise. The following is a brief outline of some such facts. To you they may be old stories, but to the majority of people in your city they would come as new and interesting information.

Tooth preparations are of three classes—powders, pastes and liquids. The first two are known as dentifrices, the last as collutoria. Powders were perhaps the earliest sort of tooth preparations. They were made then, as now, principally of chalk, magnesium carbonate, soap, flavor, sometimes artificial color, and occasionally, also, some antiseptic substance.

The chalk is the scouring and polishing agent, and should be the finest precipitated chalk, in order that it may not scratch the enamel. Sometimes prepared chalk is substituted. Prepared chalk is NOT precipitated chalk. It contains many small, gritty particles which are hard and sharp enough to cut into the teeth. It should never, therefore, be used in a dentifrice. Other substances sometimes used as abrasives are pumice stone, charcoal, orris root, etc. None of these has any place in a tooth powder. The pumice stone cannot be prevented from scratching the teeth, and charcoal, orris root and other vegetable substances will, if the slightest particle remains between the teeth, give rise to the same sort of fermentation and consequent decay that would be produced by particles of food under similar circumstances.

The light magnesium carbonate is usually employed to give bulk to the powder. It has no particular scouring or polishing ability, and its only advantage (?) is that we are deceived into believing that we are getting a large amount for our money.

The soap serves two purposes. It has a certain cleansing quality, and serves also to make the powder slightly alkaline. It is needless to say that only the best quality of soap should be used, but some manufacturers have been strongly suspected of using up their "odds and ends" in this profitable way. Even so good a thing as soap may become less valuable however, if too much of it is used. There are some otherwise excellent dentifrices on the market in which the quantity of soap is so large that they have a tendency to cause the brush to simply slip over the teeth, instead of scouring them, and thus much of the good effect of the precipitated chalk is lost. Too much soap may almost be considered as an adulteration.

The flavor of a tooth preparation is of greater importance than might appear at first thought. A dentifrice with a disagreeable taste is about as useless as one which contains something actually harmful. Few people can be induced to properly clean the teeth when the cleansing process involves the use of an ill-tasting mixture.

Artificial coloring is used solely to attract the eye. It adds nothing to the value of the powder and should be omitted, though it does no particular harm.

The value of antiseptics in tooth powders is very problematical. They do not remain in the mouth long enough to enter into complete solution, and certainly not long enough to render it sterile unless the quantity of antiseptic is abnormally large. One of the chief benefits of an antiseptic— to the manufacturer— is that it furnishes an excellent opportunity for some attractive advertising, and this feature is not neglected. It is even overdone, and some statements are made which would not look well in the light of a little accurate knowledge. For example, one manufacturer prints on his box this statement: "———— ———— tooth powder stands alone as an ideal tooth powder containing ALL the valuable germ destroying antiseptics." Just analyze this statement for yourselves and make your own comments.

There should be a very sharp distinction drawn between

*flavor* and antiseptic quality. Too often these are confused and the flavor is regarded as playing a large part in the cleansing process.

Briefly, then, the good tooth powder will contain:

(1) Precipitated chalk, because this will scour the teeth without scratching them.

(2) A moderate quantity of good soap, to aid in the cleansing process and to render the powder slightly alkaline.

(3) A flavoring material of pleasant taste to promote its thorough and frequent use.

(4) No such substances as prepared chalk, pumice stone, orris root, charcoal, mineral acids (except boric acid), mercury or potassium salts, salicylic acid or salol, or others which might have a harmful effect on the tooth structure.

After the powders came the liquid preparations. Evidently these cannot scratch the teeth, and they have been frequently advertised as possessing this desirable quality. This is indeed true, but a danger as bad as scratching lurks in some of them. Several on the market are slightly acid in reaction, and the harmfulness of this need not be discussed. Many more are mere frauds, containing nothing except water in which a little soap has been dissolved, and which has been highly colored and flavored. Ivory soap and water would be a thousand times more desirable as a tooth cleanser. One manufacturer charges fifty cents a bottle for a mixture composed mainly of tannin and water, and advertises it as possessing wonderful qualities. It should be distinctly understood that to purchase the ordinary tooth liquid is a waste of good money that cannot be too severely frowned upon. I do not, mind you, refer here to gargles, but to those tooth liquids which are supposed to act merely as tooth cleansers.

These pastes are very similar to the powders. Indeed, they are practically the same thing, made into a paste by means of glycerine and gelatin. They possess the same good qualities, and if bad are open to the same objections. In fact, a scratching paste is worse than a too abrasive powder, because it clings more closely to the brush and comes into rather more intimate contact with the teeth.

Elaborate chemical analyses are not necessary. to determine the suitability of the ordinary dentifrice to its purpose. Simple qualitative tests, combined, if desired, with microscopical examination, furnish all the information needed. Such tests may be similar to the following:

(1) *Scratching.* Place some of the sample upon a microscopic slide, moisten with water and cover with another slide. Rub the two slides together freely with this thin paste between them. Wash and dry the slides. Hold to the light and examine for scratches.

The slides are made of extremely hard glass, and any tooth powder or paste which will scratch glass under the above conditions should not be used for the purpose of an ordinary dentifrice. If a powder or paste feels gritty between the teeth, it should certainly be subjected to this test, carefully made at once.

(2) *Alkalinity.* Shake a small amount of the powder or paste with water, and add a drop or two of phenolphthalein solution (made by dissolving phenolphthalein in alcohol). A basic reaction is shown by the appearance of a bright pink color, and the preparation which will not produce this reaction should be rejected.

(3) *Soap.* Shake a small portion of the sample with warm water. The amount of foam produced is a very rough indication of the quantity of soap present.

(4) *Carbonate.* A few drops of hydrochloric acid added to the preparation will, by the effervescence, indicate the presence of a carbonate. If it is desired to carry this test further, the usual chemical tests for calcium and magnesium may be made.

(5) *Microscopic Examination.* For the dentist the microscope furnishes the quickest and at the same time a very satisfactory means for the detection of harmful substances, especially in powders. Comparison with ingredients of known purity will quickly show the presence of anything deleterious.

In discussing this question I have spoken very frankly, and perhaps rather strongly at times, because I believe that it is the duty and the privilege of the Massachusetts Dental Society to take a much more active part than it has in the past in disseminating accurate information regarding this and other important branches of oral hygiene. There are several very definite lines of activity which, if undertaken generally and carried out conscientiously, would, I believe, result in very great good to the people of Massachusetts. With your permission, I will, in closing, suggest one or two such avenues of effort.

(1) *Take your patients into your confidence.* Talk English to them and do not simply befog their minds with technical terms which mean nothing to them. Advise them definitely, and tell them *why*, being very certain that your own explanation will stand the light of day.

(2) *Encourage and work actively for the spread of accurate information in the schools.* This is being done to some extent, but not actively enough. See to it that such instruction is given in high schools as well as in the grades—and to the teachers as well as the pupils. It might not be out of place to hold meetings in the school for parents, just as the doctors are doing in connection with tuberculosis.

(3) *Frame and secure the passage of a law requiring the manufacturers of all dental preparations to print upon the label their complete formula (including proportions).* In no other way can the public be better safeguarded against inferior dentifrices. Make *all* do what some of the more reliable are doing at the present time—state honestly the composition of their preparations. Make it possible for the dentist to recommend dentifrices which may be bought in open market and still not violate that excellent statement of your Committee on Preparations, that "no profession should be called *learned* that uses or prescribes secret remedial and prophylactic preparations." This Society has some influence at the State House. That has been very evident during the last few weeks. Use that influence to secure something even more important to the people than the appointment of a satisfactory examiner.

If you can do this one thing alone for our protection, I believe you will have done a work worthy of comparison with the fight of physicians against the white plague, and I believe that you will gain for the Massachusetts Dental Society a place of respect among the people of this State that no other society enjoys.—*Journal of Allied Societies*, for June 1913.

